# Fronto-limbic neural variability as a transdiagnostic correlate of emotion dysregulation

**DOI:** 10.1038/s41398-021-01666-3

**Published:** 2021-10-21

**Authors:** Valeria Kebets, Pauline Favre, Josselin Houenou, Mircea Polosan, Nader Perroud, Jean-Michel Aubry, Dimitri Van De Ville, Camille Piguet

**Affiliations:** 1 Department of Psychiatry, University of Geneva, Geneva, Switzerland; 2 Department of Radiology and Medical Informatics, University of Geneva, Geneva, Switzerland; 3 Department of Electrical and Computer Engineering, National University of Singapore, Singapore, Singapore; 4 Laboratoire Neuro-psychiatrie, AP-HP, Département Médico-Universitaire de Psychiatrie et d’Addictologie (DMU IMPACT), Hôpital Henri Mondor, Fondation FondaMental, Université Paris Est Créteil (UPEC), INSERM U955, IMRB, 94010 Créteil, France; 5 Université Paris Saclay, CEA, UNIACT, Neurospin, 91191 Gif-sur-Yvette, France; 6 Department of Psychiatry and Neurology, CHU Grenoble Alpes, Univ. Grenoble Alpes, Inserm, U1216, GIN, 38000 Grenoble, France; 7 Institute of Bioengineering/Center for Neuroprosthetics, Ecole Polytechnique Fédérale de Lausanne (EPFL), Lausanne, Switzerland

**Keywords:** Neuroscience, Psychiatric disorders, Biomarkers

## Abstract

Emotion dysregulation is central to the development and maintenance of psychopathology, and is common across many psychiatric disorders. Neurobiological models of emotion dysregulation involve the fronto-limbic brain network, including in particular the amygdala and prefrontal cortex (PFC). Neural variability has recently been suggested as an index of cognitive flexibility. We hypothesized that within-subject neural variability in the fronto-limbic network would be related to inter-individual variation in emotion dysregulation in the context of low affective control. In a multi-site cohort (*N* = 166, 93 females) of healthy individuals and individuals with emotional dysregulation (attention deficit/hyperactivity disorder (ADHD), bipolar disorder (BD), and borderline personality disorder (BPD)), we applied partial least squares (PLS), a multivariate data-driven technique, to derive latent components yielding maximal covariance between blood-oxygen level-dependent (BOLD) signal variability at rest and emotion dysregulation, as expressed by affective lability, depression and mania scores. PLS revealed one significant latent component (*r* = 0.62, *p* = 0.044), whereby greater emotion dysregulation was associated with increased neural variability in the amygdala, hippocampus, ventromedial, dorsomedial and dorsolateral PFC, insula and motor cortex, and decreased neural variability in occipital regions. This spatial pattern bears a striking resemblance to the fronto-limbic network, which is thought to subserve emotion regulation, and is impaired in individuals with ADHD, BD, and BPD. Our work supports emotion dysregulation as a transdiagnostic dimension with neurobiological underpinnings that transcend diagnostic boundaries, and adds evidence to neural variability being a relevant proxy of neural efficiency.

## Introduction

Emotion regulation allows individuals to modulate, manage or organize emotions in order to help them meet the demands of the environment and achieve their goals [1–3], implicating various processes and systems (e.g., cognitive, behavioral, social, biological). In contrast, emotion dysregulation has been described as “a pattern of emotional experience and/or expression that interferes with appropriate goal-directed behavior” [4]. Emotion dysregulation is a central feature of psychopathology, and is key to both the development and maintenance of mood, personality and anxiety disorders, among others [5–7]. Because it is both a risk factor for psychopathology in the general population, and is common across many forms of psychiatric disorders, a better understanding of the neurobiological underpinnings of emotion dysregulation may have important clinical applications, for instance in predicting or measuring the efficacy of a therapeutic intervention [8, 9] in a transdiagnostic setting.

Emotion regulation is thought to rely on a fronto-limbic network, whereby the prefrontal cortex (PFC) exerts cognitive control over the amygdala, a subcortical structure that is central to emotion processing and salience perception [10–12]. Unsurprisingly, alterations in this network are central to the pathophysiology of bipolar disorder (BD), borderline personality disorder (BPD), and attention deficit/hyperactivity disorder (ADHD), which are all characterized by emotion dysregulation [13–18]. Notably, the three disorders also share risk factors, such as childhood trauma and genetic overlap [19–22]. This suggests that emotion dysregulation might have underlying neurobiological mechanisms that are shared across these disorders.

A measure that has received increasing attention in the past few years is neural variability, obtained by computing within-subject BOLD signal variability over the timecourse. First considered as neural “noise”, it has since been proposed as an index of local system dynamics [23]. Indeed, a certain level of instability is thought to be required for the brain to flexibly explore different functional network configurations and adapt to various environmental demands [24–26]. Neural variability has been shown to vary with age [27–32], task performance [28, 30, 33, 34], but also symptom severity [35–38]. However, these relationships are often not linear (e.g., inverted U-shape in development and aging), and are task-, difficulty-, and circuit-dependent [33, 39, 40]. Nevertheless, this body of work demonstrates the functional relevance of neural variability, and that it can provide meaningful information that is complementary to mean-based measures.

Studies investigating neural variability in clinical populations have implicated neural circuits that are relevant for psychopathology. Indeed, brain signal variability in the medial PFC during rest was shown to correlate positively with increased ADHD symptoms and inattention in children with and without ADHD [38]. Furthermore, brain signal variability has been shown to vary with mood shifts. In patients with BD, opposing patterns of neural variability were found in the default and sensorimotor networks (SMN) between patients in the depressed and manic phases [37]. This pattern mirrored the psychomotor behavior (i.e., acceleration/slowing), as well as the affective state (i.e., external/internal focus) that characterize the manic and depressive phases, respectively. Similarly, higher brain signal variability in the SMN was shown in individuals with a cyclothymic temperament compared to those with a depressive temperament in the general population [35]. Interestingly, it was suggested that increased neural variability in specific circuits might facilitate local neuronal responses to incoming stimuli, and lead to over-excitation of specific behaviors/symptoms, e.g., psychomotor behavior, ruminations [35, 37]. However, to date, most studies looking at neural variability have relied on case–control comparisons, and few have tested for transnosographic, dimensional relationships.

In contrast to traditional case–control reports, a recent movement in psychiatry has advocated for a dimensional approach in the search for neurobiological markers of psychiatric symptoms. The NIMH’s Research Domain Criteria (RDoC) framework is one of the initiatives working towards developing a neurobiologically-based classification of mental disorders that integrates findings from behavioral science, neuroscience, and genetics [41, 42]. Consequently, we favored a transdiagnostic approach in the present work by leveraging a multi-site cohort of healthy individuals and individuals suffering from conditions strongly associated with emotion dysregulation (ADHD, BD, and BPD). We aimed to identify an emotion dysregulation dimension with associated patterns of blood-oxygen level-dependent (BOLD) variability, present in varying degrees among all individuals from our transdiagnostic cohort, which would suggest common neurobiological mechanisms that transcend diagnostic boundaries. We relied on partial least squares, a multivariate data-driven technique that extracts latent components by maximizing covariance between spatial patterns of neural variability and behavior (here, emotion dysregulation, as expressed by a combination of affective lability, depression and mania assessments). More specifically, we hypothesized that neural variability in the fronto-limbic circuit would be related to individual variation in emotion dysregulation.

## Materials and methods

### Participants

Data for this study were collected from three sites (Geneva, Paris, Grenoble; see Fig. S1 for participants’ inclusion and exclusion criteria). All participants gave their written informed consent. The research was conducted according to the principles of the Declaration of Helsinki and was approved by the University of Geneva research Ethics Committee (CER 13–081), the Paris CPP Ile de France IX Ethics Committee, and the Grenoble University Hospital Ethics Committee (n° 2011-A00425-36). Inclusion criteria for all participants were age between 18 and 55, no history of alcohol or drug abuse/dependence, no current or past cardiac or neurological disease. Exclusion criteria for all participants were a history of neurological disease or head trauma with loss of consciousness, any significant cerebral anatomic abnormality, and contraindications for MRI.

Individuals with BD were recruited from the outpatient Mood Disorder Program of the Geneva University Hospital, from two university-affiliated participating centers (AP-HP, Henri Mondor Hospitals Créteil and Fernand Widal-Lariboisière Hospitals, Paris, France), and from the expert center for BD of Grenoble University Hospital. The clinical diagnosis was established using the DSM-IV-TR criteria by specialized psychiatrists and confirmed by the Mini-International Neuropsychiatric Interview [43], the Structured Clinical Interview for the DSM-IV [44], or the Diagnostic Interview for Genetic Studies (DIGS) [45]. Individuals were under stable medication for four weeks. Patients in Grenoble and Geneva were included in the study if they reported having been euthymic for at least 1 month prior to scanning and if they had a MADRS score <15 and a YMRS score <7. Patients in Paris were not in the acute phase of BD at the time of scanning.

Individuals with BPD and ADHD were recruited from the outpatient Emotional Dysregulation Unit for BPD and ADHD of the Geneva University Hospital. BPD diagnosis was established with the SCID for DSM-IV Axis II Personality Disorders [46], and ADHD diagnosis with the Diagnostic Interview for ADHD in Adults (DIVA 2.0), by trained clinicians as part of the standard procedure of these specialized programs. Some patients were under psychotropic medication for comorbidities, as reported in Table 1. Participants were instructed not to take psychostimulants on the day of the study data acquisition.

**Table 1:** Demographic, imaging, and clinical profile of the sample used in the analyses (*N* = 166).

	Bipolar (*N* = 75)	ADHD (*N* = 20)	Borderline (*N* = 21)	Controls (*N* = 65)	*F*/chi²	*p* val
*Demographics*						
Age, mean (SD)	37.22 (11.61)	24.00 (3.45)	27.05 (4.67)	35.06 (12.01)	10.82	**1.61E−06**
Sex (F/M)	30/33	7/13	19/0	37/27	20.39	**1.41E−04**
Education, mean (SD)^a^	13.22 (2.70)	16.00 (2.81)	14.84 (3.18)	12.67 (2.86)	8.34	**3.97E−05**
*Imaging*						
Scanner (1/2/3/4)	16/21/10/16	20/0/0/0	19/0/0/0	16/48/0/0	68.21	**1.03E−14**
Framewise displacement, mean (SD)	0.17 (0.09)	0.14 (0.05)	0.13 (0.04)	0.17 (0.07)	7.59	**8.83E−05**
*Clinical*						
ALS, mean (SD)	1.04 (0.64)	1.12 (0.48)	1.80 (0.46)	0.42 (0.40)	39.44	**3.44E−19**
MADRS, mean (SD)	4.84 (4.51)	3.55 (3.44)	7.68 (3.45)	1.23 (2.17)	20.90	**1.69E−11**
YMRS, mean (SD)	1.68 (1.87)	0.00 (0.00)	1.47 (1.39)	0.66 (1.39)	8.77	**2.00E−05**
*Disease severity*						
Disease duration, mean (SD)^b^	14.59 (9.34)	6.80 (5.31)	9.23 (5.39)	–	–	–
# Hospitalizations, mean (SD)^c^	3.95 (3.24)	0.05 (0.22)	1.94 (2.54)	–	–	–
*Medication (by target)*^d^						
Dopaminergic, No. (%)	44 (70%)	15 (75%)	1 (5%)	0 (0%)	–	–
Serotonergic, No. (%)	46 (73%)	1 (5%)	2 (11%)	0 (0%)	–	–
Glutamatergic, No. (%)	42 (67%)	0 (0%)	0 (0%)	0 (0%)	–	–
GABAergic, No. (%)	36 (57%)	0 (0%)	0 (0%)	0 (0%)	–	–
Norepinephrinergic, No. (%)	30 (48%)	15 (75%)	0 (0%)	0 (0%)	–	–
Lithium, No. (%)	38 (60%)	0 (0%)	0 (0%)	0 (0%)	–	–
No medication, No. (%)	30 (48%)	5 (25%)	17 (89%)	46 (72%)	–	–
Medication load, mean (SD)	2.14 (1.51)	0.85 (0.59)	0.16 (0.50)	0.31 (0.53)	–	–

Groups were compared with either ANOVAs (for continuous measures) or chi-squared tests (for categorical measures). All *p*-values that survived false discovery rate (FDR) correction (*q* < 0.05) are indicated in bold. Disease severity and medication use are only shown for informative purposes, but were not compared between groups.

^a^Based on 138 participants.

^b^Based on 82 patients.

^c^Based on 55 patients.

^d^Medication was sorted by the neurotransmitter system(s) affected by the medication used by participants, based on the Neuroscience-based Nomenclature (NbN-2 [91, 92], http://nbn2r.com/). The list of medications and their categorization can be found in Table S5. Note that percentages may add up to more than 100% because some individuals take more than one medication.

Control participants were recruited via local databases as well as through web advertisement and were matched with patients in terms of age, sex, level of education, and handedness. Exclusion criteria were past or present neurological or psychiatric disorders (Geneva, Grenoble), personal or family history of Axis I mood disorder, schizophrenia, or schizoaffective disorder (Paris), use of psychotropic medication, and contraindication for MRI. All participants underwent clinical assessment by trained raters using the DIGS [45].

In total, 122 individuals with BD were recruited on all three sites, 93 healthy controls (HC) were recruited on two sites (i.e., Geneva and Paris), while 24 individuals with BPD and 21 individuals with ADHD were only recruited on one site (i.e., Geneva). We excluded ten participants (8 BD, 1 BPD, and 1 HC) for excessive in-scanner motion; 69 participants (39 BD, 2 BPD, 1 ADHD, and 27 HC) because they had not completed the clinical measures of interest; nine participants because they scored above 15 on the MADRS (7 BD, 2 BPD) and 6 because they scored above 7 on the YMRS (5 BD, 1 HC). The final sample thus comprised 166 participants, including 63 euthymic BD, 20 ADHD, 19 BPD, and 64 HC. The demographic, imaging, and clinical data of the final sample are shown in Table 1.

### Clinical assessment

We used the Affective Lability Scales (ALS [47]), the Montgomery-Åsberg Depression Rating Scale (MADRS [48]), and the Young Mania Rating Scale (YMRS [49]) to measure different facets of emotion dysregulation. The ALS specifically measures affective lability, which refers to the frequency, speed, and range of changes in affective states [50]. The ALS is a 54-item self-reported questionnaire on which participants rate the tendency of their mood to shift between a “normal state” and different affects (depression, anger, anxiety, and elation), as well as their tendency to experience shifts between elation and depression, and between anxiety and depression. The total score was obtained by averaging across the six subscales, i.e., anger, anxiety, anxiety/depression, depression, depression/elation, elation. The MADRS and YMRS are both clinician-rated scales that evaluate depressive and manic symptoms, respectively. The total score (sum across all items) was used for both scales.

### Magnetic resonance imaging acquisition

Briefly, participants were scanned on 3T MRI scanners (see detailed MRI acquisition parameters in the Supplementary Methods). A resting state (RS) functional magnetic resonance imaging (fMRI) sequence, as well as an anatomical scan were acquired in all participants.

### Resting state fMRI preprocessing

The first ten RS functional images were discarded to ensure signal equilibration, and the remaining images were preprocessed using SPM12 tools (http://fil.ion.ucl.ac.uk/spm/software/spm12). Functional images were first realigned, followed by co-registration of the mean functional image with the anatomical scan. Functional images were normalized to the MNI space with SPM12 “Segment”, resampled to 3 mm isotropic voxels, and then spatially smoothed with a 6 mm full-width-at-half-maximum (FWHM) Gaussian filter. The average signal within a mask of white matter (WM) and cerebrospinal fluid (CSF) were extracted using the Data Processing Assistant for Resting-State fMRI toolbox [51]. The effects of WM, CSF, and six motion parameters were regressed out from the time-course, and a bandpass filter (0.01–0.10 Hz) was applied. Motion scrubbing [52] was applied to correct for motion artefacts; i.e., framewise displacement (FD) was calculated as the sum of the absolute values of the six realignment parameters, and scans with a FD higher than 0.5 mm, as well as one scan before and two scans after, were excluded from the analysis. Participants with a time-course containing less than 4 min of scanning were excluded (8 BD, 1 BPD, and 1 HC).

### DARTEL group template

A group template was generated with the Diffeomorphic Anatomical Registration Through Exponentiated Lie Algebra (DARTEL [53]) from the gray matter and white matter tissue segments of all the participants comprising the entire original sample (*N* = 250, see Fig. S1). Participants’ T1 images were first segmented using the Computational Anatomy Toolbox (CAT12; http://www.neuro.uni-jena.de/cat/) “Segment Data” and the tissue segments were normalized to the tissue probability maps by means of an affine transformation. The group template was then normalized to the MNI space, and additional registration to the ICBM template was applied. Finally, the template was downsampled in order to match the dimensions of the functional images, and then binarized to include only voxels with a ≥50% gray matter probability.

Because of incomplete cerebellar coverage in 33 participants, we decided to exclude the cerebellum from the DARTEL template. To do so, we used a bilateral mask of the cerebellum as defined in Hammers atlas [54, 55], a probabilistic anatomical atlas based on 83 manually-delineated regions drawn on MR images of 30 healthy adult subjects. In order to encompass the whole cerebellum, we first smoothed the mask with a 25 mm FWHM Gaussian filter, then downsampled the mask to match the dimensions of the DARTEL template, and excluded the cerebellum mask from the DARTEL template.

### BOLD signal variability

Voxel-wise BOLD signal variability was obtained for each participant by computing the standard deviation of each preprocessed time-course. This approach is equivalent to the frequency-domain computation of the amplitude of low-frequency fluctuations (ALFF) between 0.01 and 0.1 Hz [56, 57], and is strongly correlated with mean-square successive differences (MSSD) [28, 58]. BOLD signal variability maps were constrained to the binarized DARTEL template (excluding the cerebellum), and were *z*-scored across all voxels included in the template within each participant [57]. Age, sex, scanner, and head motion (i.e., mean FD) were linearly regressed out from the imaging data prior to the PLS analysis using a general linear model on MATLAB.

### Partial least squares (PLS) analysis

We used PLS analysis to identify BOLD signal variability spatial patterns related to emotion dysregulation across all participants. PLS is a multivariate data-driven statistical technique that aims to maximize covariance between two matrices [59, 60]. The optimal relationship between the two data matrices is represented as latent components (LCs), which are weighted linear combinations of the original data that maximally covary with each other. A LC is characterized by a spatial pattern of neural variability and a behavioral pattern of affective lability, depression, and mania (imaging and behavioral saliences, respectively). By linearly projecting the imaging and behavioral measures of each participant onto their respective saliences, we obtain individual-specific brain and behavior scores, which reflect the participants’ imaging and behavioral contribution to each LC. Importantly, the PLS analysis was agnostic on the diagnostic group, so that transdiagnostic brain-behavior associations could be extracted.

The statistical significance of the LCs was assessed by constructing a null distribution of the singular values using permutation testing (1000 permutations), whereby the behavioral data was permuted within each diagnostic group, so that latent components would not be driven by group differences. To determine which behavioral measures and voxels were driving the significant LC, we computed Pearson’s correlations between the original imaging data and brain scores, as well as between the original behavioral measures and behavioral scores [61, 62]. A higher positive (or negative) correlation for a particular behavioral measure for a given LC indicates greater importance of the behavioral measure for the LC, while a higher positive (or negative) correlation for a particular imaging measure for a given LC indicates greater importance of that imaging value for the LC. We estimated confidence intervals for these correlations with a bootstrapping procedure that generated 1000 samples with replacement from participants’ imaging and behavioral data, while accounting for diagnostic groups (i.e., bootstrap resampling was performed within each diagnostic group) in order to avoid spatial and behavioral patterns being driven by group differences, since our aim was to find transdiagnostic patterns of emotion dysregulation. *Z*-scores were computed by dividing each correlation coefficient by its bootstrap-estimated standard deviation, and were considered as strong contributors to LCs at absolute values >3, corresponding to a robustness at a confidence interval of approximately 99% [60]. See Supplementary Methods for more details.

### Posthoc analyses

Two-sample *t*-tests were performed to test whether brain and behavioral scores were different between participants from different diagnostic groups. Group differences in demographics, head motion, and clinical measures were tested using one-way analysis of variance (ANOVA; for continuous measures) or chi-squared tests (for categorical measures). We also tested if there were any significant associations between PLS brain (or behavioral) scores and disease severity, as well as medication use, using either Pearson’s correlations (for continuous measures), or *t*-tests (for binary measures). All posthoc analyses were corrected for multiple comparisons at a false discovery rate (FDR) of *q* < 0.05.

### Control analyses

A number of control analyses were computed to assess the robustness of our results (detailed in the Supplementary Methods). Briefly, we used BOLD signal variability maps that included the cerebellum; we accounted for education level, early life trauma, or disease severity; we considered patients only; and we considered only participants from the one site that included individuals from all four diagnostic groups to control for scan effects. Finally, group differences and the impact of disease severity on the brain-behavior associations were examined.

## Results

### Neural variability correlates of emotion dysregulation

**Fig. 1:**
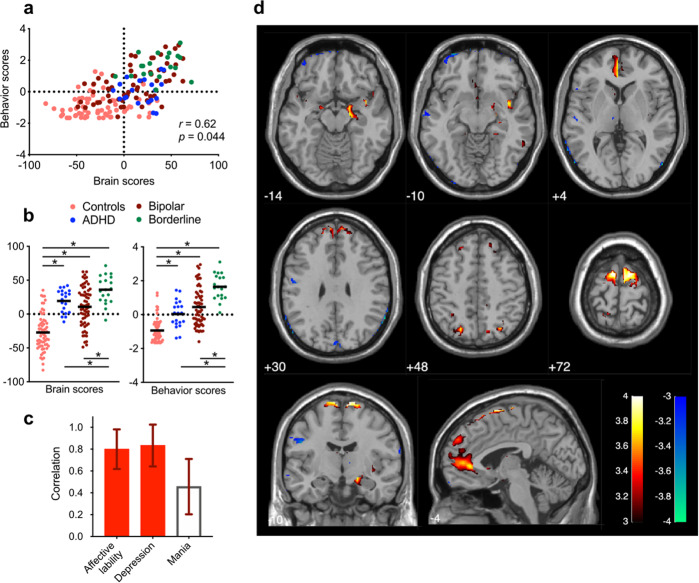
The first latent component (LC1) is characterized by high emotion dysregulation. **a** PLS correlation between individual brain and behavior scores for LC1. Each dot is a participant from any of the four diagnostic groups. **b** Group differences in brain and behavior scores for LC1. Bold lines are mean scores for each group, while asterisks indicate two-sample *t*-tests that have survived to FDR correction (*q* < 0.05). Controls have significantly lower brain and behavior scores compared to all patient groups. BPD patients have significantly higher brain and behavior scores compared to ADHD and BD patients. **c** Greater depression and affective lability characterize the behavioral pattern of LC1 (see Table S1). Mania did not have a strong contribution to LC1 (*z* < 3). Loadings are Pearson’s correlations between participants’ original behavioral data and their behavior scores, and error bars indicate bootstrap-estimated standard deviations. **d** LC1 is characterized by increased BOLD signal variability in the left ventromedial PFC, bilateral dorsomedial PFC, subgenual ACC, bilateral amygdala, right hippocampus, right insula, and bilateral motor cortex, as well as decreased BOLD signal variability in occipital regions (see Table S2 for MNI coordinates of peaks of all significant clusters). Loadings are *z*-scores obtained from bootstrapping, thresholded at absolute values ≥3 (*p* < 0.01).

We applied PLS to whole-brain BOLD signal variability and a combination of clinical measures that characterize emotion dysregulation in 166 participants that were either healthy or had a diagnosis of ADHD, BD, or BPD. PLS revealed one significant latent component (LC1). LC1 revealed a significant association (*r* = 0.62, *p* = 0.044) between BOLD signal variability and emotion dysregulation (Fig. 1a), accounting for 74% of the covariance between the two matrices.

Healthy individuals had significantly lower brain and behavior scores compared to all patient groups (Fig. 1b). Moreover, individuals with BPD had significantly higher brain and behavior scores compared to individuals with ADHD and BD. This dimension was therefore expressed more strongly by individuals with BPD, and less strongly by individuals with no psychiatric diagnosis, although there was a large overlap among individuals from all diagnostic groups (as shown on Fig. 1a).

Higher loadings on LC1 were associated with greater affective lability and depression, whereas mania did not yield a strong contribution (Fig. 1c and Table S1). On the imaging side, LC1 was characterized by increased brain signal variability in the left ventromedial PFC, bilateral dorsomedial PFC, subgenual ACC, bilateral amygdala, right hippocampus, bilateral motor cortex, and right insula, as well as decreased variability in occipital regions (Fig. 1d and Table S2 for the list of all reliable peaks and their MNI coordinates). This pattern partly recapitulates the emotion regulation network, which is known to be dysfunctional in ADHD, BD, and BPD [13–18]. We note however that LC1 also featured brain regions whose role in emotion dysregulation is not clear (e.g., occipital regions).

Post-hoc associations between brain (or behavior scores) and disease severity, as well as medication use, can be found in Table 2 and Table S3. Using medication affecting the serotonergic system was associated with higher behavioral scores. When categorizing medication use by medication class, the use of stimulants was also associated with higher brain scores.

**Table 2:** Post-hoc associations between participants’ brain (or behavioral) scores, disease severity, and medication use (categorized by the neurotransmitter target).

	Brain scores		Behavior scores	
	*r*/*t*	*p*	*r*/*t*	*p*
*Disease severity*				
Disease duration	0.11	0.334	0.01	0.958
# Hospitalizations	−0.16	0.253	0.16	0.244
*Medication use (by target)*				
Dopaminergic	2.40	0.018	2.10	0.037
Serotonergic	−0.10	0.924	2.61	**0.010**
Glutamatergic	−0.18	0.860	−0.04	0.969
GABAergic	−0.81	0.417	1.46	0.146
Norepinephrinergic	2.25	0.026	0.89	0.376
Lithium	−1.87	0.064	0.73	0.467
No medication	0.99	0.325	−0.74	0.458
# Meds	−0.12	0.153	0.14	0.090

Pearson correlations (for continuous measures) or t-tests (for categorical measures) were computed across all participants. A higher *r* value indicates a stronger association between brain (or behavioral) scores and disease severity, while a higher *t* value indicates higher brain (or behavioral) scores in participants taking the specific medication. Significant correlations or *t*-tests that survived FDR correction (*q* > 0.05) are indicated in bold. The same analysis was performed after classifying medication by medication class (see Table S3).

### Control analyses

Control analyses whereby we i) used BOLD signal variability maps including the cerebellum; ii) accounted for education level or early life trauma; iii) considered patients only (i.e., excluded control participants); and iv) considered only participants from one site, all yielded saliences that were similar to the original brain and behavior saliences (see Table S4), all showing the reliability of our findings. Detailed results are reported in the Supplementary Results, Tables S5–S7, and Figs. S2–S4.

## Discussion

In this work, we aimed to identify spatial patterns of neural variability related to emotion dysregulation in a multi-site transdiagnostic cohort, using a multivariate data-driven approach. We found that emotion dysregulation was associated with a pattern of increased BOLD signal variability in the ventromedial PFC, dorsomedial and dorsolateral PFC, amygdala, hippocampus, insula and motor cortex, and decreased BOLD signal variability in occipital regions. Our findings are in line with emotion dysregulation being a dimensional construct that spans across individuals with affective disorders such as ADHD, BD, and BPD, rather than being specific to any of these disorders. Importantly, the spatial pattern of brain signal variability associated with this dimension bears a compelling resemblance to the fronto-limbic circuit that is thought to subserve emotion regulation, and is impaired in ADHD, BD, and BPD. Our findings therefore add evidence to brain signal variability being a relevant proxy of neural efficiency, and support emotion dysregulation as a transdiagnostic dimension with neurobiological underpinnings that transcend diagnostic boundaries.

The patterns of brain signal variability associated with greater emotion dysregulation were mainly located in the fronto-limbic system, which plays a key role in emotional control/regulation [12, 63]. Critically, abnormalities in the fronto-limbic network are thought to underpin emotion dysregulation in pathophysiological models of BD and BPD [13–16, 18]. The suggested mechanism involves hyper-activation of limbic regions responsible for emotion generation—in particular, the amygdala, hippocampus, and ventral striatum-, coupled with hypo-activation of the PFC, which is responsible for cognitive control. This circuit has shown structural abnormalities in individuals with BD [14, 64–66], BPD [15, 16], but also ADHD [67], e.g., altered volumes of the amygdala and hippocampus, and cortical thinning of the PFC. In BPD patients, abnormal patterns of activity in the amygdala, hippocampus, ventrolateral PFC, and dorsolateral PFC were shown during emotion processing [15, 16, 68], but also at rest, where the ACC, medial PFC and dorsolateral PFC were found to be hyper-activated during resting state in BPD patients compared to control participants [69]. Furthermore, neural activation and connectivity of fronto-limbic regions, especially the ACC, amygdala, insula, and ventrolateral PFC, showed changes following psychotherapy aimed at improving emotion regulation in BPD patients [70]. Abnormal patterns of activity and connectivity of fronto-limbic regions have been reported in euthymic BD patients, especially involving the amygdala and the medial PFC at rest [71, 72], and during emotion regulation tasks [73, 74]. Moreover, psychosocial intervention in individuals with BD or at risk for BD may induce functional and structural changes in these regions [75–77]. Emotion dysregulation is also prevalent in ADHD, and fronto-limbic alterations involving the amygdala, orbitofrontal cortex, ventral striatum, and PFC have also been reported in this population [17]. We note however, that in addition to fronto-limbic regions, spatial patterns of neural variability maps associated with LC1 also featured brain regions whose role in emotion dysregulation is not clear (e.g., occipital regions; see Table S3 for a list of the BOLD signal variability clusters that reliably contributed to LC1).

Previous studies of neural variability in ADHD [38, 78–80], BD [37, 81–86], and BPD patients [87, 88] have failed to show any consistent pattern, although some have reported alterations in regions of the fronto-limbic network (mostly the PFC). In ADHD patients, increased BOLD signal variability in the dorsolateral PFC, inferior frontal and orbitofrontal cortex were found during a Stroop task [78], as well as increased brain signal variability in the ventromedial PFC during a vigilance task in adolescents with ADHD compared to controls [80]. Moreover, greater MSSD in the dorsomedial PFC during rest was related to greater ADHD symptom severity, while greater MSSD in the ventromedial PFC was positively correlated with inattention across children with ADHD and typically developing children [38]. In BPD patients, increased ALFF was shown in the hippocampus [88], while increased ALFF in the ventral PFC, dorsolateral PFC, and insula were found in euthymic BD patients [85], compared to controls. The fronto-limbic circuit also overlaps with the DMN, in particular the ventromedial PFC and hippocampus. We found increased neural variability of the ventromedial PFC to be associated with LC1, which was mostly driven by greater levels of depression. This partly corroborates a previous study contrasting neural variability patterns in the DMN and SMN, i.e., higher DMN/SMN ratio in the depressive phase of BD and the inverse pattern during mania, which were positively correlated with depression and mania scores, respectively [37]. Therefore, our findings somewhat corroborate previous reports of altered neural variability in these clinical populations, but for the first time in a network directly associated with emotion regulation.

Our findings may be in apparent contrast to the prevailing view that brain signal variability facilitates neural flexibility by allowing fluid transitions between brain states via a stochastic resonance effect [24, 25]. However, while it appears to be beneficial to task performance, heightened neural variability was also shown to correlate with worse clinical symptoms in various conditions [36–38]. As neural variability is thought to increase sensitivity to incoming stimuli, it is possible that heightened neural variability might lead to over-reactivity of specific neural circuits, as a maladaptive strategy to prepare for potentially relevant events, which instead supports the maintenance of emotion dysregulation in affective disorders. Consequently, our findings support the use of neural variability as a relevant proxy for dysfunctional emotional processing, which might be useful in tracking symptom severity and treatment efficacy [89].

The present study has several strengths including the use of a multivariate data-driven approach and the relatively large transdiagnostic cohort. Moreover, our approach aligns with recent initiatives such as the RDoC [41, 42], that promote neurobiologically-based approaches to investigate dimensions of (ab)normal functioning, which often transcend classical nosological categories. Computational techniques may help in this endeavor by deriving brain-behavior associations that could serve as potential phenotypes. Moreover, by quantifying their neurobiological correlates, data-driven machine learning approaches such as PLS could help refine future taxonomies for mental disorders.

However, this work also has several limitations. First, because of poor cerebellar coverage in a number of participants, we decided to exclude the cerebellum from our analyses, even though an increasing number of reports implicate this structure in higher-order processes. Moreover, the diagnostic groups were not balanced across the three sites, which is a common shortcoming in multi-site studies with different protocols. Most patients were also using psychotropic medication at the time of scanning, which is known to modulate intrinsic brain activity metrics [90]. Our posthoc analyses showed mild associations (*t* < 3) between LC1 and medication use. Some control analyses were only exploratory, as sample sizes were small because of missing data (e.g., *N* = 55 and *N* = 82 when accounting for hospitalizations and disease duration, respectively). While PLS is a powerful technique for identifying brain-behavior associations, they remain correlational and do not claim to imply any causal relationship. Disease severity effects may have impacted our findings, in particular the number of hospitalizations which yielded high loadings when added to the clinical variables (see Supplementary Results), however we note that our cohort was not extensively phenotyped to investigate this question in depth, as many patients were missing this information (e.g., *N* = 55 when accounting for hospitalizations). Finally, some of our control analyses suggest that LC1 might be partly driven by group differences between the patient groups and control participants. Future studies are needed to replicate and further investigate these effects.

Despite some limitations, our findings have unveiled the neural variability correlates of emotion dysregulation in the fronto-limbic system, further improving our understanding of the pathogenesis of affective disorders. Importantly, emotion dysregulation is a transdiagnostic construct that has shown clinical utility as a therapeutic target, as demonstrated by a decrease in maladaptive emotion regulation strategy use and symptom severity (e.g., depression, anxiety, substance use, etc.), regardless of the treatment protocol, the construct of emotion regulation that was examined, and the targeted disorder [9]. Indeed, transdiagnostic protocols aimed at improving emotion regulation have been shown to provide rapid and significant improvement in individuals with various forms of severe mental illness [8]. In this context, our approach might also provide a robust way of tracking therapeutic effects of interventions aimed at enhancing emotion regulation.

## Supplementary information

Supplemental Material

## Data Availability

The code for the MRI preprocessing, BOLD signal variability extraction, as well as the PLS outputs can be found on https://github.com/valkebets/BOLDsd_ED, while the code for the PLS analysis is publicly available at https://github.com/danizoeller/myPLS.
