# Supplementary material for: Fronto-limbic neural variability as a transdiagnostic correlate of emotion dysregulation
Source: Transl Psychiatry. 2021 Oct 21;11:545. doi: 10.1038/s41398-021-01666-3 (PMC8530999; doi:10.1038/s41398-021-01666-3)
Supplement: Supplementary file 1 — Supplemental Material [file 41398_2021_1666_MOESM1_ESM.docx]

**Fronto-limbic neural variability as a**

**transdiagnostic correlate of emotion dysregulation**

***Supplemental Information***

Supplementary Methods

MRI acquisition parameters

Geneva (Site 1)

MRI data were acquired at the University of Geneva on a 3T Siemens MAGNETOM TrioTim scanner with a 32-channel head coil. A resting state functional magnetic resonance imaging (rs-fMRI) sequence as well as an anatomical T1-weighted scan were acquired. Whole brain functional images were collected using a BOLD-weighted EPI sequence (TR/TE = 2100/30 ms; flip angle = 80 degrees; PAT factor = 2; FOV = 205 mm; matrix size = 64 x 64 pixels). Thirty-six transversal slices were acquired sequentially with a 3.2 mm thickness and an interslice gap of 3.84 mm, yielding a voxel size of 3.2 x 3.2 x 3.2 mm. 250 volumes were acquired for a total duration of 8 minutes 45 seconds. Subjects were instructed to close their eyes and let their thoughts wander, and not to fall asleep. A high-resolution whole brain anatomical scan was acquired with a T1-weighted 3D sequence (MPRAGE; TR/TI/TE = 1900/900/2.27 ms; flip angle = 9 degrees; voxel dimensions = 1.0 mm isotropic).

Paris (Site 2)

MRI data were collected at Neurospin (Commissariat à l’Energie Atomique, Saclay, France) using a 3T Siemens Magnetom TrioTim with a 12-channel head coil. The rs-fMRI images were collected using the following acquisition parameters: EPI sequence; TR/TE = 2000/27 ms; flip angle = 81 degrees, FOV = 192 mm, voxel size = 3 x 3 x 3 mm. 360 volumes were acquired for a total duration of 12 minutes. Participants were instructed to close their eyes and try not to think about anything specific, without falling asleep. A high-resolution T1-weighted anatomical scan was also acquired (MPRAGE; TR/TI/TE = 2300/900/2.98 ms; flip angle = 9 degrees; voxel dimensions = 1 x 1 x 1.1 mm).

Grenoble (Sites 3 and 4)

Imaging data were acquired using two whole-body 3T MR scanners (Bruker MedSpec S300 and Achieva 3.0 TX Philips) at the Grenoble MRI facility IRMaGE with a similar fMRI acquisition sequence. Sixteen and 28 patients with BD were scanned on the first and second scanner, respectively. Functional images were acquired using the following acquisition parameters for both scanners: interleaved EPI sequence, T2*-weighted; TR/TE = 2500/30 ms; flip angle = 77 degrees; FOV = 216 mm; matrix size = 72 x 72 pixels). Thirty-nine slices were acquired transversally on the Bruker scanner, with a 3.5 mm thickness, and an interslice gap of 3 mm, yielding a voxel size of 3 x 3 x 3.5 mm. Thirty-seven slices were acquired transversally on the Philips scanner with a 3 mm thickness, an interslice gap of 3 mm, and a voxel size of 3 x 3 x 3.75 mm. volumes were acquired on both scanners for a total duration of 6 minutes. Subjects were asked to stay awake with their eyes open and to not think about anything in particular. A high-resolution whole brain anatomical scan was acquired with a T1-weighted 3D sequence (TR/TI/TE = 1900/900/2.27 ms; flip angle = 9 degrees; voxel dimensions = 0.8 mm isotropic; acquisition matrix 280 x 320 x 220 pixels). A 3D Modified Driven Equilibrium Fourier Transform (MDEFT) and a turbo field echo (TFE) sequence were used for acquisitions using the Bruker and the Philips scanners, respectively.

Medication use

Medication was categorized by the affected neurotransmitter system, according to the Neuroscience-based Nomenclature (NbN-2 ^1,2^, <https://nbn2r.com/>). Targeted neurotransmitters included the dopaminergic, GABAergic, glutamatergic, lithium, norepinephrinergic, and serotonergic. Posthoc analyses involving the use of “other” medication were not tested, because these medications were very heterogeneous and not necessarily psychotropic. In a control analysis, we also classified medication by medication class (e.g., antidepressants, mood stabilizers, etc.). The list of all medications used by participants, and their categorization into targeted neurotransmitter(s) and medication class can be found in **Table S5**.

Partial least squares analysis

Partial least squares (PLS) is a multivariate data-driven statistical technique that aims to extract latent components (also called latent variables), representing optimal brain-behavior associations ^3,4^. While it is mechanistically similar to canonical correlation analysis (CCA), PLS has the advantage of not suffering from rank deficiency when the number of features is higher than the number of sample, or in case of high autocorrelation (e.g., spatial autocorrelation or correlation between behavioral variables), which makes it ideally suited for neuroimaging data ^5^.

BOLD signal variability maps are stored in $X$ (participants $\times$ voxels), while the clinical data are stored in $Y$ (participants $\times$ behavioral measures). After z-scoring both matrices across all participants, we computed a cross-covariance matrix $R$:

$$R =Y^{T}X$$

followed by singular value decomposition of $R$, which provides the reconstruction of $R$ by matrices $U$, $S$, and $V$:

$$R =USV^{T}$$

The singular vectors $V$ and $U$ are called the *brain* and *behavior* *saliences* (akin to loadings in principal components analysis), while $S$ is a diagonal matrix containing the singular values. Next, we computed $L_{X}$ and $L_{Y}$ by projecting $X$ and $Y$ onto their respective saliences $V$ and $U$**:**

$$L_{X} =XV$$

$$L_{Y} =YU$$

The matrices obtained (one for the imaging data and one for the behavior data, per LC) are called *brain* and *behavior scores* (akin to the scores in principal components analysis) and reflect the participants’ imaging and behavioral contribution to each LC.

The covariance explained by each LC is estimated by dividing the squared singular value by the sum of all squared singular values, and the LCs are ordered by the amount of covariance they account for.

The contribution of the original variables to the LC relied on *structure coefficients*, i.e., Pearson’s correlations between $X$ and $L_{X}$, as well as between $Y$ and $L_{Y}$ ^6,7^. Structure coefficients are commonly used in factor analysis, multiple regression analysis and CCA, as they help to define the structure of the synthetic variables, and thereby reflect the direct contribution of a predictor to the predictor criterion (independently of other predictors), which can be critical when predictors are highly correlated between each other (i.e., in presence of multicollinearity ^8^). Notably, structure coefficients were high correlated to saliences in our data (*r* = 0.90, *r* = 1 with brain and behavior saliences of LC1, respectively).

Control analyses

A number of control analyses were performed to assess the robustness of our results, whereby we examined the similarity (i.e., Pearson correlation) between the saliences (i.e., brain and behavioral) obtained in the original PLS model and the saliences obtained in the control analyses. First, since recent reports have implicated the cerebellum in emotion and cognitive processes ^9–11^, the PLS analysis was re-computed after including the cerebellum in BOLD signal variability maps (*N* = 133), after excluding participants with incomplete cerebellar coverage. Second, we re-ran the analysis in participants with available education level data (*N* = 138), after adding it to the other regressors. Furthermore, there is substantial evidence showing that early life trauma impacts the clinical expression of mood disorders ^12^; therefore, we re-computed the analysis in participants who were administered the Childhood Trauma Questionnaire (*N* = 138) after adding its total score to the regressors. Because scanner can crucially impact imaging measures, we re-ran our analysis by including only participants from Geneva (*N* = 71), which was the only site that included individuals from all four diagnostic groups.

Group differences in the brain-behavior association

We performed several analyses to test whether the LCs were driven by group differences (between controls and patients, or between patient groups). First, the PLS analysis was re-computed only in patients (*N* = 102). Second, we removed group means from data of each diagnostic group prior to the PLS analysis, by z-scoring both imaging and behavioral data *within* groups instead of *across* all participants. Third, we performed a group behavior PLS analysis whereby a separate covariance matrix was computed for each diagnostic group, resulting in a separate set of behavior saliences for each group, which are related to a common BOLD signal variability pattern. Fourth, we computed a task mean-centered PLS model, whereby instead of the behavioral measures, the $Y$ matrix coded for the diagnostic groups.

Impact of disease severity on the brain-behavior association

We examined the impact of disease severity on LC1 in three different ways. First, we tested if there were any significant associations between PLS brain (or behavioral) scores and disease severity across all participants, and within each patient group. Next, we re-computed the PLS analysis after regressing out disease duration and number of hospitalizations from the imaging data, in order to see if the LC1 was still significant. Finally, we re-computed the PLS analysis after adding disease duration and number of hospitalizations to the clinical variables, and checked if the two variables yielded high loadings, thus impacting the brain-behavior association.

Supplementary Results

Control analyses

Including the cerebellum in BOLD signal variability maps (*N* = 133) yielded very similar patterns (Pearson *r* = 0.94, *r* = 1 with the original brain and behavior saliences of LC1, respectively; see **Table S4**). After accounting for education level (*N* = 138), the brain-behavior association remained significant (*p* = 0.006), and very similar to the original results (*r* = 0.65, *r* = 0.90 with the original brain and behavior saliences, respectively). When accounting for early life trauma (*N* = 138), results were moderately similar to the original analysis (*r* = -0.54, *r* = 0.40 with the original brain and behavior saliences, respectively). To account for scanner effects, we re-ran permutation testing (i.e., a null distribution of the singular values) accounting for scanner, and found that LC1 remained significant (*p* = 0.021). We also computed another control analysis considering participants from only one site (Geneva; *N* = 71) as it was the only site that included individuals from all four diagnostic groups, and we found moderate to very high similarity with the original results (*r* = -0.50, *r* = 0.98 with brain and behavior saliences, respectively).

Group differences in the brain-behavior association

When considering only patients (i.e., excluding control participants from the analysis), results were very similar to the original analysis (*r* = 0.65, *r* = 1 with the original brain and behavior saliences, respectively; see **Table S4**).

When the data were z-scored within each diagnostic group to remove group means prior to the PLS analysis, LC1 was no longer significant (*p* = 0.078, after permuting data *within* groups). When we examined the saliences, we found identical patterns (*r* = 1 for both behavior and imaging saliences) to the saliences of our original model (**Table S4**, **Figure S2**). This suggests that while the brain-behavior association we have found is present after removing group differences, the association is weakened after removing these group differences.

Next, we computed a group behavior PLS analysis, whereby a separate covariance matrix was computed for each diagnostic group, resulting in a separate set of behavior saliences for each group, which are related to a common BOLD signal variability pattern. LC1 was no longer significant (*p* = 0.132), and there was no LC that resembled the transdiagnostic component that we found (**Figure S3**).

Additionally, we computed a mean-centered task PLS analysis to formally test for an association between BOLD signal variability and diagnostic group. We found a significant LC (*p* = 0.001, explaining 72% of the covariance), which contrasted the three patient groups vs. the control participants (**Figure S4**). The imaging saliences were very similar to the loadings reported in our main analysis (Pearson *r* = 0.91; **Table S4**), suggesting that the brain-behavior association (i.e., LC1) from our main model might indeed be driven by group differences, in particular differences between the patient groups and control participants. Notably, the three patient groups yield very similar saliences, which suggests that we captured shared effects of emotion dysregulation across individuals with bipolar disorder, ADHD, and borderline personality disorder.

Impact of disease severity on the brain-behavior association

We ran exploratory analyses to investigate disease severity effects on LC1. First, we tested for associations between brain/behavioral scores and disease duration/number of hospitalizations in all participants with available information, and found no significant correlation (**Table 2**). We further tested these effects *within each diagnostic group* that had these data available (i.e., all patient groups), and found a very different picture. Indeed, while individuals with bipolar disorder and borderline personality disorder generally showed strong *positive* correlations between their brain/behavioral scores and disease severity, individuals with ADHD showed the reverse pattern (i.e., *negative* correlation with disease severity); see **Tables S6** and **S7**. This suggests that there are disease severity effects, which might impact different groups differently.

Next, we regressed out disease severity from the imaging data, and found that LC1 was no longer significant (*p* = 0.568 in the model adjusted for disease duration; *p* = 0.161 in the model adjusted for number of hospitalizations). However, this may be due to the low sample size in which the disease severity data were available (*N* = 82 and *N* = 55). Therefore, to test whether this was due to the low sample size, we computed our main PLS analysis in the two subsamples where information about disease duration and number of hospitalizations were available (*N* = 82 and *N* = 55, respectively). LC1 was not significant in any of the two models, although close to significance in the second model (*N* = 82, *p* = 0.568; *N* = 55, *p* = 0.063).

Finally, we re-computed the PLS analysis after adding disease duration and number of hospitalizations to the clinical variables. On the one hand, the PLS model with number of hospitalizations was significant (LC1 *p* = 0.012), and the number of hospitalizations yielded a strong loading (*r* = 0.64) – the second highest contribution after MADRS (*r* = 0.79), and higher than the contribution of ALS (*r* = 0.55) and YMRS (*r* = 0.47). On the other hand, the PLS model with disease duration was not significant (LC1 *p* = 0.667), and disease severity had a low loading (*r* = -0.13) compared to other clinical variables (ALS *r* = 0.89, MADRS *r* = 0.49, YMRS *r* = 0.47).

Therefore, these control analyses suggest that the number of hospitalizations affects the brain-behavior association that we found. However, we note that our cohort was not extensively phenotyped to explore this question in depth, as some of these subsamples were small (i.e., *N* = 82 and *N* = 55 when accounting for disease duration and number of hospitalizations, respectively).

**Table S1. Contribution of each behavioral measure to LC1.** Pearson’s correlations between participants’ behavioral measures and their behavioral scores, along with their bootstrap-estimated standard deviations (SD) are shown. Absolute *Z* scores above 3 were considered reliable (*p* > 0.01) and are indicated in bold.

|  |  |  |
| --- | --- | --- |
| **Behavior variable** | **Correlation (SD)** | **Z-score** |
|  |  |  |
| Affective lability (ALS) | 0.89 (0.18) | **4.40** |
| Depression (MADRS) | 0.83 (0.19) | **4.35** |
| Mania (YMRS) | 0.46 (0.25) | 1.80 |
|  |  |  |
|  |  |  |

**Table S2**. **Anatomical regions and MNI coordinates of BOLD signal variability clusters that reliably contributed to LC1** (absolute z-scores ≥ 3). Anatomical labels were determined using the Anatomy toolbox (v.3) in SPM12, which applies probabilistic algorithms to determine the cytoarchitectonic labeling of MNI coordinates ^13–15^.

| **Anatomical region** | **Cluster size** | **MNI coordinates** | | | **Z-score** |
| --- | --- | --- | --- | --- | --- |
|  |  | **x** | **y** | **z** |  |
|  |  |  |  |  |  |
| *Positive clusters* |  |  |  |  |  |
| L Superior parietal lobule / Postcentral gyrus | 326 | -12 | -7 | 77 | 3.99 |
| R Hippocampus / Parahippocampal gyrus | 247 | 21 | -16 | -13 | 3.37 |
| R Superior frontal gyrus / Precentral gyrus | 220 | 12 | -1 | 74 | 3.70 |
| L Frontal pole / Paracingulate gyrus | 208 | -3 | 56 | 5 | 3.74 |
| R Superior frontal gyrus / Postcentral gyrus | 126 | 15 | -34 | 80 | 3.51 |
| R Frontal pole / Superior frontal gyrus | 124 | 18 | 56 | 35 | 3.38 |
| L Frontal pole / Superior frontal gyrus | 92 | -21 | 44 | 38 | 3.25 |
| R Insular cortex / Planum polare | 83 | 36 | -13 | 5 | 3.35 |
| L Anterior cingulate gyrus / Paracingulate gyrus | 67 | -3 | -4 | 41 | 3.13 |
| R Temporal pole / Frontal orbital cortex | 62 | 45 | 17 | -16 | 2.97 |
| L Superior frontal gyrus / Paracingulate gyrus | 39 | -3 | 50 | 35 | 2.80 |
| R Middle temporal gyrus / Inferior temporal gyrus | 36 | 60 | -52 | -7 | 2.94 |
| R Inferior temporal gyrus / Temporal occipital fusiform cortex | 35 | 51 | -49 | -19 | 3.49 |
| L Insular cortex / Planum polare | 30 | -36 | 8 | -16 | 2.90 |
| L Superior lateral occipital cortex | 28 | -21 | -70 | 44 | 3.08 |
| R Superior lateral occipital cortex | 26 | 27 | -67 | 47 | 3.19 |
| R Temporal fusiform cortex / Inferior temporal gyrus | 22 | 30 | -1 | -49 | 2.72 |
| L Temporal pole | 20 | -51 | 11 | -13 | 2.72 |
|  |  |  |  |  |  |
| *Negative clusters* |  |  |  |  |  |
| L Inferior lateral occipital cortex / Occipital pole | 659 | -60 | -67 | 20 | 3.91 |
| L Frontal pole / Frontal medial cortex | 245 | -30 | 65 | -10 | 3.59 |
| R Frontal pole | 165 | 42 | 56 | -13 | 3.74 |
| L Postcentral gyrus / Precentral gyrus | 165 | -48 | -13 | 32 | 3.81 |
| R Cuneal cortex / Occipital pole | 162 | 3 | -88 | 29 | 3.21 |
| R Angular gyrus / Supramarginal gyrus | 147 | 60 | -61 | 29 | 3.67 |
| L Middle temporal gyrus | 133 | -54 | -13 | -16 | 3.33 |
| R Postcentral gyrus / Precentral gyrus | 89 | 69 | -7 | 17 | 3.56 |
| L Planum temporale / Heschl's gyrus | 63 | -54 | -19 | 5 | 3.27 |
| L Putamen / Frontal orbital cortex | 60 | -27 | 11 | -7 | 2.78 |
| L Inferior temporal gyrus | 58 | -54 | -37 | -28 | 3.83 |
| R Occipital pole / Inferior lateral occipital cortex | 55 | 36 | -94 | -7 | 3.02 |
| L Frontal orbital cortex / Frontal operculum cortex | 44 | -30 | 26 | -7 | 3.19 |
| R Frontal orbital cortex / Subcallosal cortex | 39 | 9 | 17 | -25 | 3.44 |
| L Frontal orbital cortex / Subcallosal cortex | 35 | -9 | 14 | -25 | 2.96 |
| R Inferior lateral occipital cortex | 35 | 54 | -76 | 5 | 3.31 |
| L Middle frontal gyrus / Inferior frontal gyrus, pars opercularis | 33 | -39 | 11 | 26 | 3.05 |
| L Middle temporal gyrus | 23 | -54 | -49 | 2 | 3.20 |
| R Inferior temporal gyrus | 20 | 54 | -28 | -31 | 2.86 |
|  |  |  |  |  |  |
|  |  |  |  |  |  |

**Table S3.** Post-hoc associations between participations’ brain (or behavioral) scores and medication use (categorized by medication class). T-tests were used to compare brain (or behavioral scores) between participants that used one type of medication compared to participants that didn’t use that type of medication. Significant t-tests that survived FDR correction (*q* > 0.05) are indicated in bold.

|  | Brain scores | | Behavior scores | |
| --- | --- | --- | --- | --- |
|  | *t* | *p* | *t* | *p* |
| *Medication use (by class)* |  |  |  |  |
| Typical antipsychotics | 1.38 | 0.170 | -1.69 | 0.093 |
| Atypical antipsychotics | -1.63 | 0.106 | -1.85 | 0.066 |
| Antidepressants | -0.12 | 0.902 | -2.08 | 0.039 |
| Mood stabilizers | 1.10 | 0.273 | -0.88 | 0.383 |
| Sedates/Hypnotics/Anxiolytics | 1.00 | 0.321 | -0.78 | 0.434 |
| Stimulants | -2.94 | **0.004** | -0.90 | 0.370 |
|  |  |  |  |  |

**Table S4.** Similarity (measured by Pearson correlation) between the saliences of each control analysis and the original saliences.

|  | *Including cerebellum* | *Regressing education* | *Regressing early life trauma* | *Patients only* | *Geneva only* | *Group z-scoring* | *Mean-centered task PLS* |
| --- | --- | --- | --- | --- | --- | --- | --- |
| Correlations with original behavior saliences | 1.00 | 0.90 | 0.40 | 1.00 | 0.98 | 1.00 | - |
| Correlations with original imaging saliences | 0.94 | 0.65 | -0.54 | 0.65 | -0.50 | 1.00 | 0.91 |
|  |  |  |  |  |  |  |  |

**Table S5.** **Medication sorted by targeted neurotransmitter system(s) and medication class.** Categorization by targeted neurotransmitter was based on the Neuroscience-based Nomenclature (NbN-2 ^1,2^; <https://nbn2r.com/>). Possible neurotransmitter targets were the acetylcholinergic, dopaminergic, epinephrinergic, GABAergic, glutamatergic, histaminic, lithium, norepinephrinergic, and serotonergic systems, as well as “other” (which included medication not targeting any neurotransmitter system). Medication targeting the acetylcholinergic, epinephrinergic, and histaminic systems were merged with “other” as these categories had too few participants (≤2) on their own. Medication classes comprised typical and atypical antipsychotics, antidepressants, mood stabilizers, sedatives/hypnotics/anxiolytics (SHA), stimulants, and “other”.

| Molecule / Medication | Targeted neurotransmitter(s) | Medication class |
| --- | --- | --- |
| Alimemazine / Theralene | Dopamine, Histamine | Typical antipsychotic |
| Almotriptan / Almogran | Serotonin | Antidepressant |
| Amisulpride / Solian | Dopamine | Atypical antipsychotic |
| Amlodipine / Amlor | Other | Other |
| Aripiprazole / Abilify | Dopamine, Serotonin | Atypical antipsychotic |
| Atomoxetine / Strattera | Norepinephrine | Stimulant |
| Biopros | Other | Other |
| Bupropion / Wellbutrin | Dopamine, Norepinephrine | Antidepressant |
| Buspirone | Serotonin | SHA |
| Carbamazepine / Tegretol | Glutamate | Mood stabilizer |
| Chlorpromazine / Largactil, Prazine | Dopamine, Serotonin | Typical antipsychotic |
| Citalopram / Seropram | Serotonin | Antidepressant |
| Clonazepam / Rivotril | GABA | SHA |
| Clorazepate / Potassium clorazepate | GABA | Other |
| Cyamemazine / Tercian | Dopamine, Serotonin | Typical antipsychotic |
| Dexmethylphenidate / Focalin | Dopamine, Norepinephrine | Stimulant |
| Diazepam / Valium | GABA | SHA |
| **Eschscholtia** | Other | Other |
| Escitalopram / Cipralex, Seroplex | Serotonin | Antidepressant |
| Estrogen / various birth control | Other | Other |
| Ezetimibe / Inegy | Other | Other |
| Fenofibrate | Other | Other |
| Fluoxetine / Prozac | Serotonin | Antidepressant |
| Flurazepam / Dalmadorm | GABA | SHA |
| Gabapentin / Neurontin | Glutamate | Other |
| Gestodene / various birth control | Other | Other |
| Iron, ferrous sulfate / Tardyferon | Other | Other |
| Lamotrigine / Lamictal | Glutamate | Mood stabilizer |
| Levomepromazine | Dopamine, Serotonin | Typical antipsychotic |
| Levonorgestrel / various birth control | Other | Other |
| Levothyroxine sodium | Other | Other |
| Lisdexamfetamine / Elvanse | Dopamine, Norepinephrine | Stimulant |
| Lithium / Lithiofor, Priadel, Teralithe | Lithium | Mood stabilizer |
| Lorazepam / Temesta | GABA | SHA |
| Loxapine | Dopamine, Serotonin | Typical antipsychotic |
| Melatonin / Circadin | Other | Other |
| Methylphenidate / Ritaline, Concerta | Dopamine, Norepinephrine | Stimulant |
| Mirtazapine | Serotonin, Norepinephrine | Antidepressant |
| Nicotine / Nicopass, Nicorette | Other | Other |
| Nux vomica | Other | Other |
| Olanzapine / Zyprexa | Dopamine, Serotonin | Atypical antipsychotic |
| Oxazepam / Anxiolit | GABA | SHA |
| Oxcarbazepine / Trileptal | Glutamate | Mood stabilizer |
| Paroxetine / Deroxat | Serotonin | Antidepressant |
| Saccharomyces boulardii / Perenterol | Other | Other |
| Prazepam / Lysanxia | GABA | SHA |
| Progestin / various birth control | Other | Other |
| Progestogen / various birth control | Other | Other |
| Quetiapine / Seroquel, Xeroquel | Dopamine, Serotonin | Atypical antipsychotic |
| Rabeprazole / Pariet | Other | Other |
| Risperidone / Risperdal, Risperdaloro | Dopamine, Serotonin, Norepinephrine | Atypical antipsychotic |
| Salmeterol / other drugs for obstructive airway diseases | Epinephrine | Other |
| Sertraline / Zoloft | Serotonin | Antidepressant |
| Simvastatin / Inegy | Other | Other |
| Topiramate / Topamax | Glutamate, GABA | Mood stabilizer |
| Tropatepine / Lepticur | Acetylcholine | Other |
| Valproic acid, valproate / Depakin, Depakote, Orfiril | Glutamate | Mood stabilizer |
| Valpromide / Depamide | Glutamate | Mood stabilizer |
| Venlafaxine / Effexor | Serotonin, Norepinephrine | Antidepressant |
| Verapamil | Other | Other |
| Zolmitriptan / Zomig | Serotonin | Other |
| Zolpidem / Stillnox | GABA | SHA |
| Zopiclone / Imovane | GABA | SHA |

**Table S6**. Post-hoc associations between participants’ brain (or behavioral) scores and disease duration, across all groups and within each diagnostic group. *ADHD = attention deficit/hyperactivity disorder; BD = bipolar disorder; BPD = borderline personality disorder.*

| *Disease duration* | **Brain scores** | | **Behavior scores** | |
| --- | --- | --- | --- | --- |
|  | *r / t* | *p* | *r / t* | *p* |
|  |  |  |  |  |
| All (N=82) | 0.11 | 0.334 | 0.01 | 0.958 |
| ADHD (N=10) | 0.16 | 0.661 | -0.20 | 0.578 |
| BD (N=59) | 0.21 | 0.111 | 0.04 | 0.786 |
| BPD (N=13) | 0.25 | 0.410 | 0.36 | 0.227 |
|  |  |  |  |  |

**Table S7**. Post-hoc associations (Pearson’s correlations) between participants’ brain (or behavioral) scores and number of hospitalizations, across all groups and within each diagnostic group. *Abbreviations: ADHD = attention deficit/hyperactivity disorder; BD = bipolar disorder; BPD = borderline personality disorder.*

| *# Hospitalizations* | **Brain scores** | | **Behavior scores** | |
| --- | --- | --- | --- | --- |
|  | *r* | *p* | *r* | *p* |
|  |  |  |  |  |
| All (N=55) | -0.16 | 0.253 | 0.16 | 0.244 |
| ADHD (N=20) | -0.43 | 0.060 | 0.02 | 0.943 |
| BD (N=19) | 0.41 | 0.083 | 0.26 | 0.280 |
| BPD (N=16) | 0.53 | 0.036 | 0.31 | 0.238 |
|  |  |  |  |  |

**Figure S1.** Flowchart illustrating the composition of the original sample and final sample, and the number of subjects excluded based on different criteria. *Abbreviations: ADHD = attention deficit/hyperactivity disorder; BD = bipolar disorder; BPD = borderline personality disorder; HC= healthy controls.*

**Figure S2.** Behavior and imaging loadings in behavior PLS analysis with group z-scoring. Both behavior and imaging loadings are identical to the patterns found in the main analysis (*r* = 1 for both behavior and imaging saliences). Behavior loadings are Pearson’s correlations between participants’ original behavioral data and their behavior scores, and error bars indicate bootstrap-estimated standard deviations. Imaging loadings are z-scores obtained from bootstrapping, thresholded at absolute values ≥ 3 (*p* < 0.01).


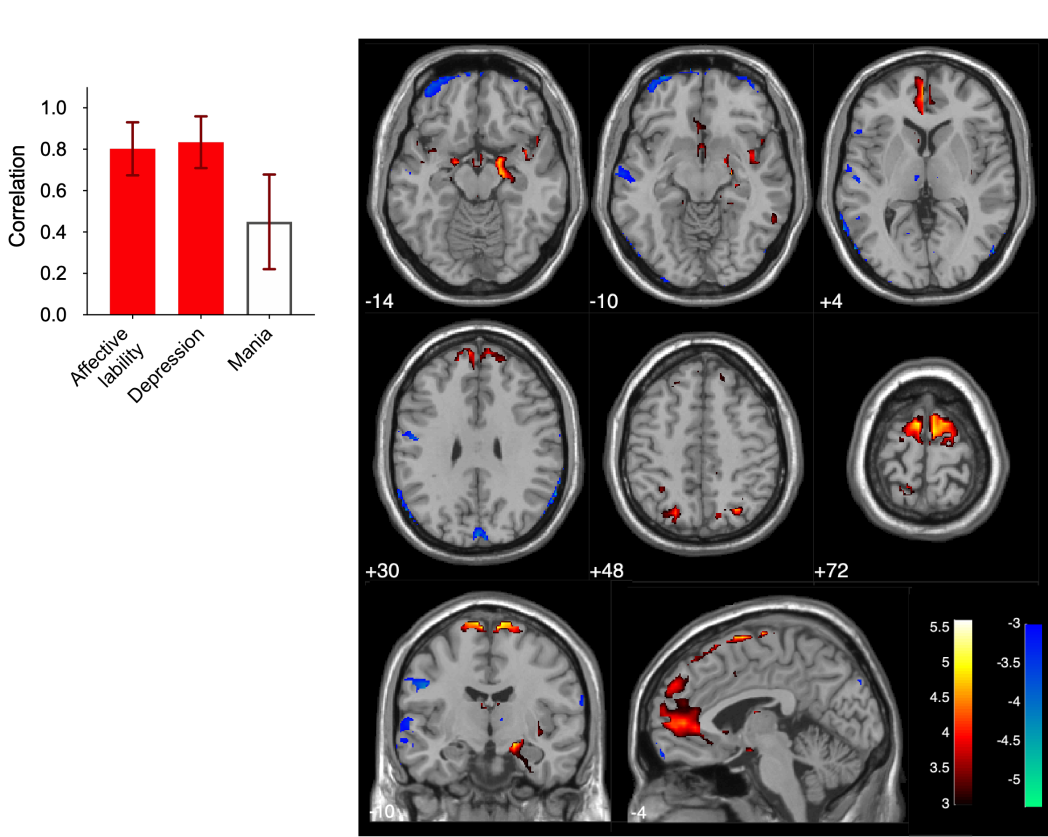


**Figure S3.** Behavior loadings of the first 3 latent components in the group behavior PLS analysis. None of the LCs were resembled LC1 from our main analysis.

**
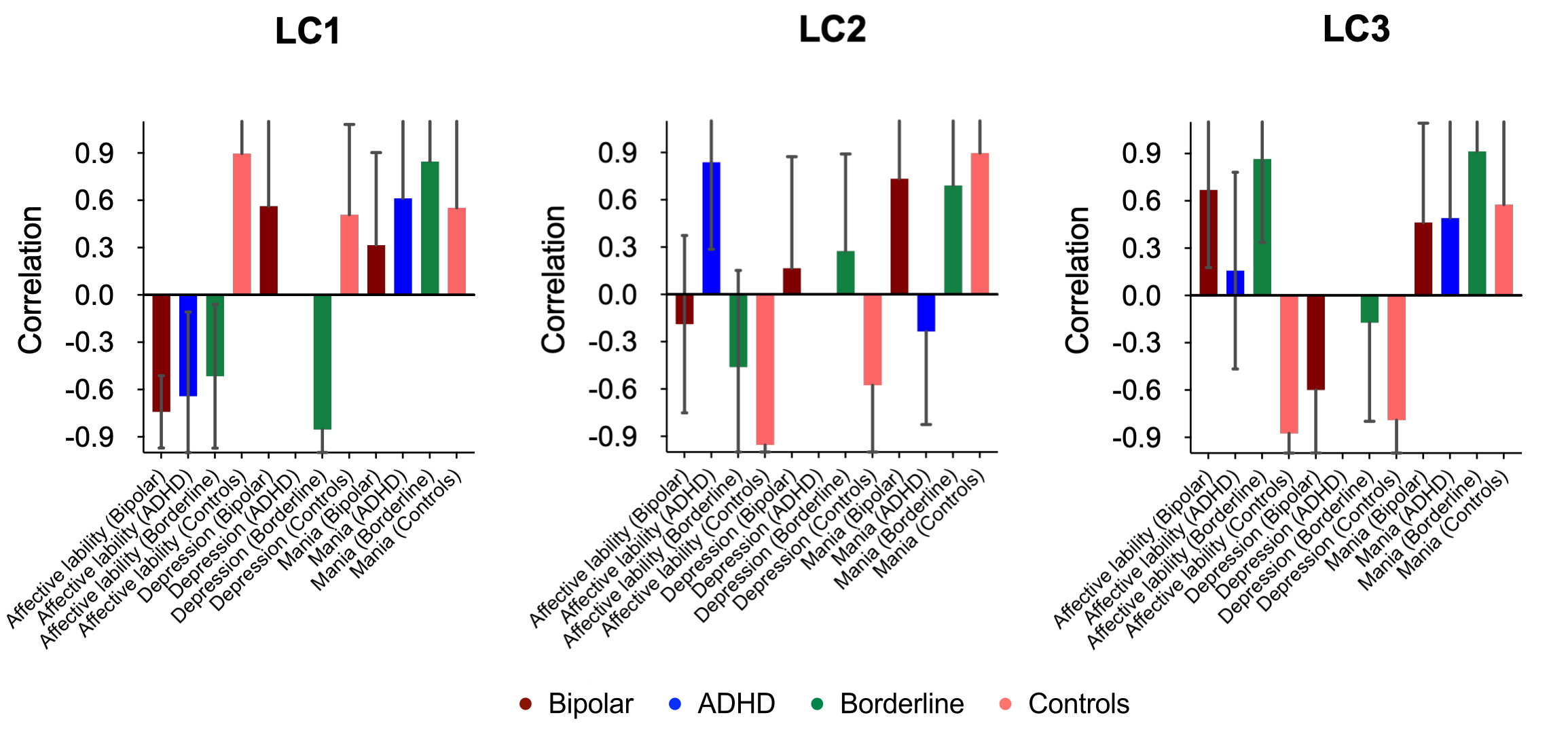
**

**Figure S4.** Behavior and imaging loadings in mean-centered task PLS analysis. In this main analysis, LC1 was significant (*p* = 0.001) and contrasted the patient groups vs. healthy controls. Imaging saliences were very similar to the salience of our main analysis (*r* = 0.91).


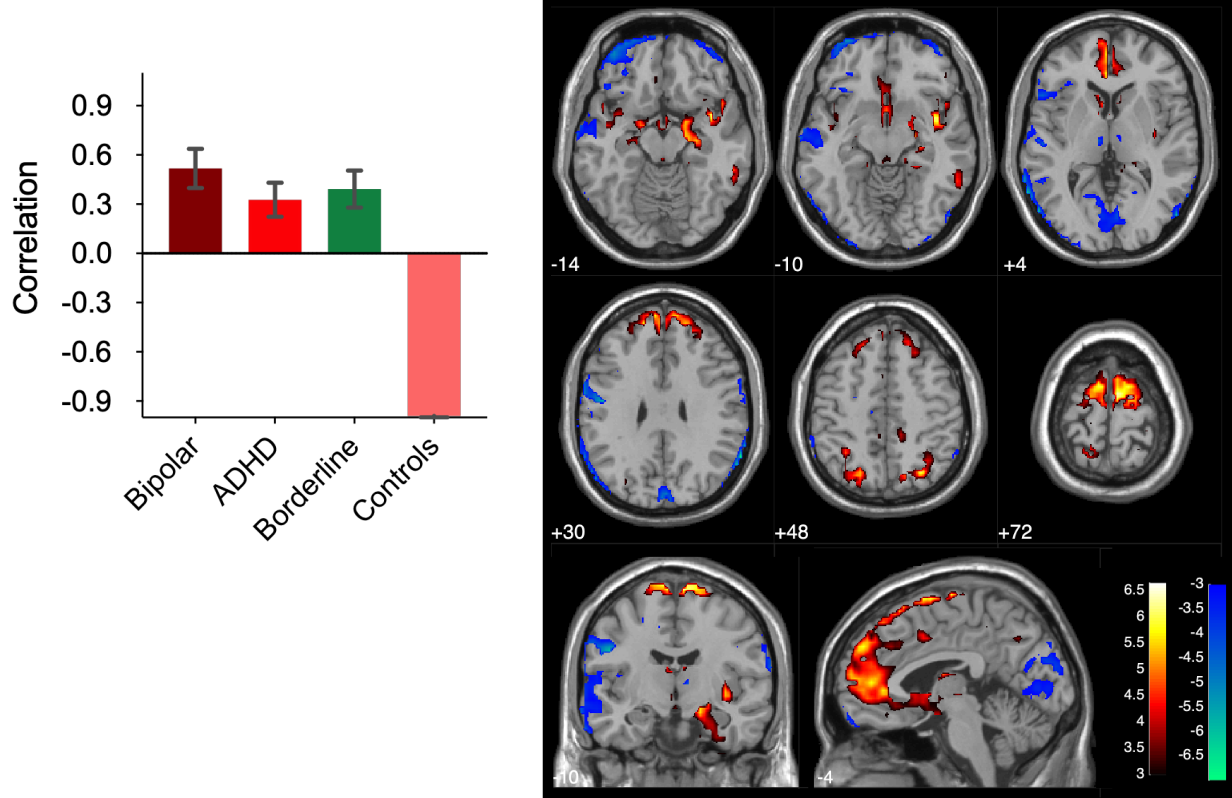


Supplementary References

1. Zohar, J. *et al.* A review of the current nomenclature for psychotropic agents and an introduction to the Neuroscience-based Nomenclature. *Eur. Neuropsychopharmacol. J. Eur. Coll. Neuropsychopharmacol.* **25**, 2318–2325 (2015).

2. Zohar, J. *et al.* A proposal for an updated neuropsychopharmacological nomenclature. *Eur. Neuropsychopharmacol. J. Eur. Coll. Neuropsychopharmacol.* **24**, 1005–1014 (2014).

3. McIntosh, A. R. & Lobaugh, N. J. Partial least squares analysis of neuroimaging data: applications and advances. *NeuroImage* **23 Suppl 1**, S250-263 (2004).

4. Krishnan, A., Williams, L. J., McIntosh, A. R. & Abdi, H. Partial Least Squares (PLS) methods for neuroimaging: a tutorial and review. *NeuroImage* **56**, 455–475 (2011).

5. McIntosh, A. R. & Mišić, B. Multivariate statistical analyses for neuroimaging data. *Annu. Rev. Psychol.* **64**, 499–525 (2013).

6. Courville, T. & Thompson, B. Use of structure coefficients in published multiple regression articles: β is not enough. *Educ. Psychol. Meas.* **61**, 229–248 (2001).

7. Henson, R. K. The Logic and Interpretation of Structure Coefficients in Multivariate General Linear Model Analyses. (2002).

8. Sherry, A. & Henson, R. K. Conducting and interpreting canonical correlation analysis in personality research: a user-friendly primer. *J. Pers. Assess.* **84**, 37–48 (2005).

9. Buckner, R. L. The Cerebellum and Cognitive Function: 25 Years of Insight from Anatomy and Neuroimaging. *Neuron* **80**, 807–815 (2013).

10. Green, M. J., Cahill, C. M. & Malhi, G. S. The cognitive and neurophysiological basis of emotion dysregulation in bipolar disorder. *J. Affect. Disord.* **103**, 29–42 (2007).

11. Moberget, T. *et al.* *Cerebellar grey matter volume is associated with cognitive function and psychopathology in adolescence*. http://biorxiv.org/lookup/doi/10.1101/288134 (2018) doi:10.1101/288134.

12. Aas, M. *et al.* Affective lability in patients with bipolar disorders is associated with high levels of childhood trauma. *Psychiatry Res.* **218**, 252–255 (2014).

13. Eickhoff, S. B. *et al.* Assignment of functional activations to probabilistic cytoarchitectonic areas revisited. *NeuroImage* **36**, 511–521 (2007).

14. Eickhoff, S. B., Heim, S., Zilles, K. & Amunts, K. Testing anatomically specified hypotheses in functional imaging using cytoarchitectonic maps. *NeuroImage* **32**, 570–582 (2006).

15. Eickhoff, S. B. *et al.* A new SPM toolbox for combining probabilistic cytoarchitectonic maps and functional imaging data. *NeuroImage* **25**, 1325–1335 (2005).
